# Research advancements on the diversity and host interaction of gut microbiota in chickens

**DOI:** 10.3389/fvets.2024.1492545

**Published:** 2024-11-19

**Authors:** Yong Yue, Pichitpon Luasiri, Jiezhang Li, Phanthipha Laosam, Papungkorn Sangsawad

**Affiliations:** ^1^School of Animal Technology and Innovation, Institute of Agricultural Technology, Suranaree University of Technology, Nakhon Ratchasima, Thailand; ^2^Postharvest Technology and Innovation in Animal Unit, Institute of Agricultural Technology, Suranaree University of Technology, Nakhon Ratchasima, Thailand; ^3^Research and Development Institute Suranaree University of Technology, Nakhon Ratchasima, Thailand

**Keywords:** gut microbiota, immune function, microbial composition, intestinal health, diseases

## Abstract

The maintenance of host health and immune function is heavily dependent on the gut microbiota. However, the precise contribution of individual microbial taxa to regulating the overall functionality of the gut microbiome remains inadequately investigated. Chickens are commonly used as models for studying poultry gut microbiota, with high-throughput 16S rRNA sequencing has emerged as a valuable tool for assessing both its composition and functionality. The interactions between the gut’s microbial community and its host significantly influence health outcomes, disease susceptibility, and various mechanisms affecting gastrointestinal function. Despite substantial research efforts, the dynamic nature of this microbial ecosystem has led to inconsistencies in findings related to chicken gut microbiota, which is largely attributed to variations in rearing conditions. Consequently, the interaction between the chickens’ gut microflora and its host remains inadequately explored. This review highlights recent advances in understanding these relationships, with a specific focus on microbial composition, diversity, functional mechanisms, and their potential implications for improving poultry production.

## 1 Introduction

The gut microbiota refers to the diverse collection of microorganisms, including bacteria, archaea, fungi, and viruses, residing in the digestive tracts of animals (1, 2). The gastrointestinal metagenome encompasses the collective genomes of these gut microorganisms (3, 4). These microbiota exert wide-ranging effects, influencing factors such as colonization resistance against pathogens, maintenance of the intestinal epithelium, metabolism of dietary and regulation of immune function, and even modulation of host behavior through the gut-brain axis (4). The microbial composition of the gut varies across different regions of the digestive tract (5), the gastrointestinal tract (GIT), which is densely populated with microorganisms that interact with both the host and ingested feed (6). In birds, the GIT contains a complex microbiota that plays a crucial role in nutrient absorption and pathogen defense (7). Specifically, in terms of pathogen defense, the gut microbiota establishes a protective barrier that limits the colonization of pathogenic bacteria (8). Additionally, dietary changes and treatment interventions have been shown to enhance poultry growth while reducing the risk of enteric infections (9). The intricate relationship between the intestinal microbiome, microorganisms, host, and diet has profound effects on both the nutrition and health of poultry (10). These interactions involve nutrient exchange, changes in gut morphology, as well as host physiology and immunity (6).

**Figure 1:**
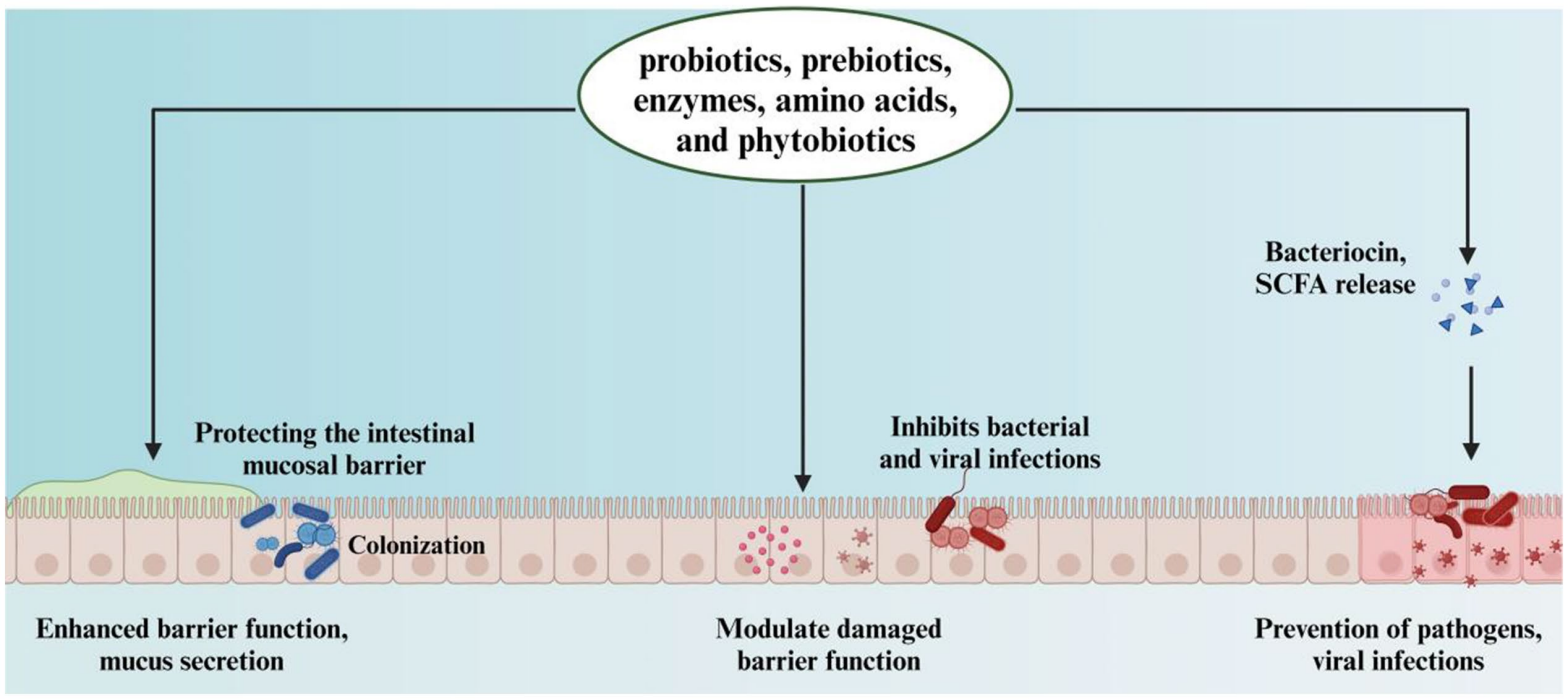
The role of probiotics, prebiotics, enzymes, amino acids, and phytobiotics in enhancing intestinal barrier and preventing infections.

Interestingly, various feed additives, including probiotics, prebiotics, enzymes, amino acids, and phytobiotics, have been demonstrated to enhance gut health (10–14). By optimizing the gut environment, these interventions lead to better feed conversion and overall health (Figure 1). Following the hatching process, the gastrointestinal tract of the chick is rapidly exposed to a diverse array of external microorganisms, fostering an optimal environment for the proliferation of anaerobic species. As these microorganisms colonize the gut, they interact with the host’s digestive processes. Over time, microbial communities diversify, and by the time chickens reach maturity, a relatively stable microbial balance is established (15). Notably, the chicken gut microbiota differs from those of other animals due to the chicken’s smaller and faster digestive tracts, resulting in a unique microbial composition (16, 17). As chickens develop, the interaction between gut microbes and host performance becomes increasingly complex (6, 18). The enzymatic degradation of indigestible dietary polysaccharides produces fermentable sugars and short-chain fatty acids (SCFAs) (19, 20). SCFAs promote the proliferation of epithelial cells in the gastrointestinal tract, thereby enhancing the surface area available for nutrient absorption (21). The bacteriostatic effects of SCFAs contribute to the elimination of foodborne pathogens, such as *Salmonella* spp. (22). Moreover, the microbiota facilitates nitrogen metabolism, incorporating nitrogen into bacterial cellular proteins. This process enables these bacteria to provide essential amino acids, proteins, and vitamins to the host (23). This review investigates the factors that influence the development of the poultry gut microbiome, with a particular emphasis on the interactions between the host and its microbiome, as well as the repercussions of microbiome disturbances on poultry health, disease susceptibility, and productivity.

## 2 Limitations and potential of 16S rRNA sequencing in microbial identification

**Figure 2:**
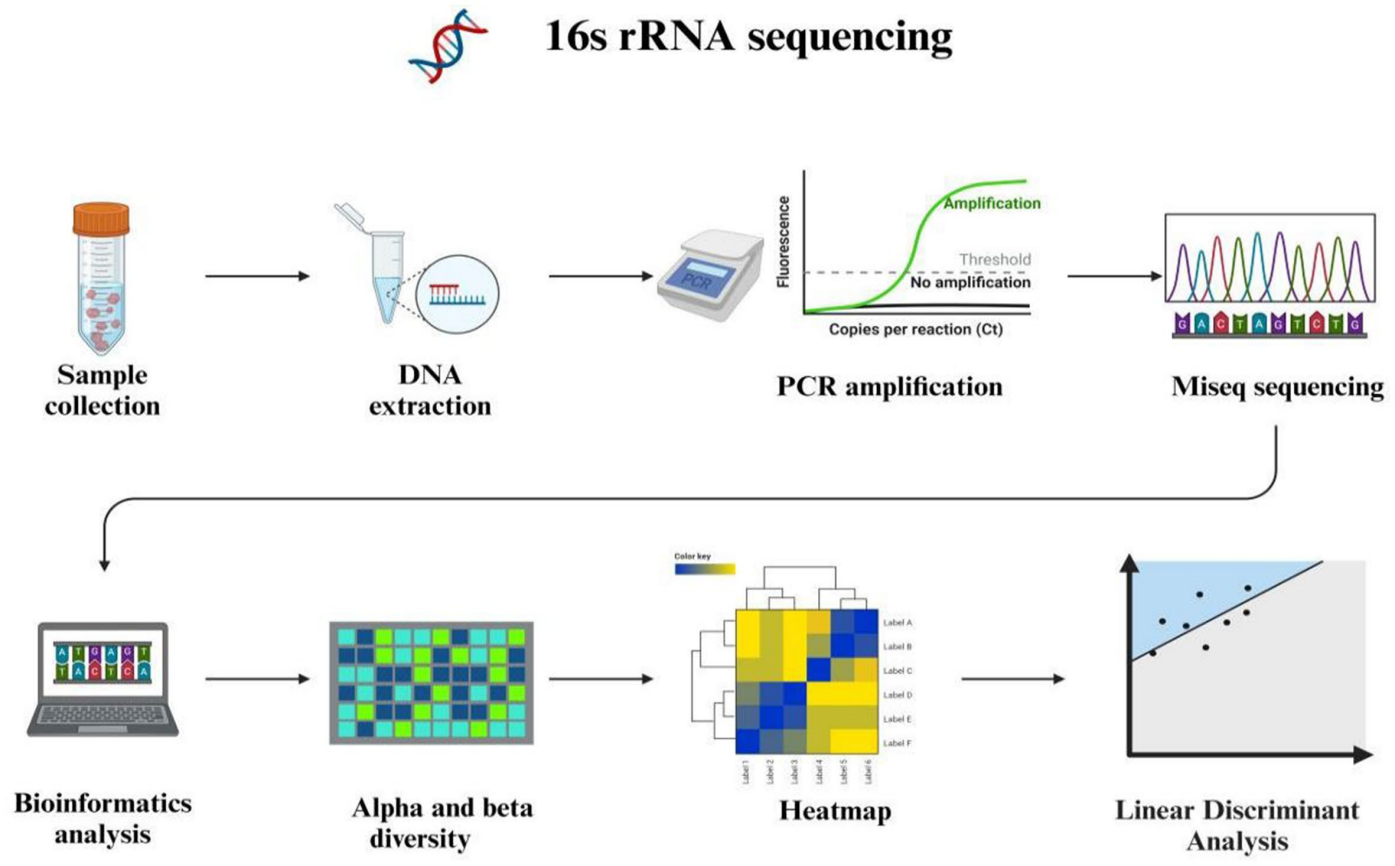
16S rRNA high throughput sequencing and bioinformatics analysis.

The 16S ribosomal RNA is widely recognized as a fundamental genetic marker for the classification and identification of bacterial species. Its ubiquitous presence across diverse bacterial taxa, conserved function in critical cellular processes, and adequate sequence length facilitate precise differentiation among various bacterial species (24). The methodology for sequencing the 16S rRNA gene targets a specific segment of microbial DNA, providing valuable insights into microbial community diversity and identification. Encoded within prokaryotic cells encompassing bacteria and archaea, the 16S rRNA gene specifies the RNA component of the 30S ribosomal subunit. Recognized as a “molecular clock” owing to its ubiquitous presence in bacteria and archaea, this gene plays an essential role in delineating phylogenetic relationships and species divergence. Leveraging the structure and functionality of the 16S rRNA gene enables researchers to trace evolutionary lineages while gaining deeper comprehension of genetic interrelationships among diverse microbial species (25). The structure of 16S rRNA comprises highly conserved segments interspersed with nine hypervariable regions facilitating concurrent sequencing of multiple species using universal primers while offering potential discrimination based solely on variable regions (26). Numerous studies have shown that 16S rRNA gene sequencing can achieve genus-level identification in over 90% of cases, but its ability to identify species-level ranges from approximately 65 to 83% (27). The Ribosomal Database Project (RDP) is a comprehensive, publicly accessible resource dedicated to the acquisition, analysis, and dissemination of ribosomal RNA (rRNA) gene sequences. It serves as an essential tool for researchers investigating microbial diversity, taxonomy, and phylogenetics. The Ribosomal Database Project (28) and SILVA databases encompass comprehensive rRNA sequence data spanning bacteria, eukarya, and archaea (29). However, 16S rRNA sequencing has several limitations (Figure 2). It does not offer insights into the metabolic potential or activity of microbial communities. The 16S rRNA gene, as a conserved housekeeping gene present in all prokaryotes, primarily reflects phylogenetic relationships rather than functional attributes such as metabolic capabilities, virulence, or antibiotic resistance. Moreover, the sequenced region of the 16S rRNA gene may lack sufficient variability to distinguish between closely related species or strains, for instance, differentiating between pathogenic and commensal strains of *Escherichia coli*.

## 3 Composition and function of the gut microbiota in chickens

### 3.1 Composition of the gut microbiota in chickens

**Figure 3:**
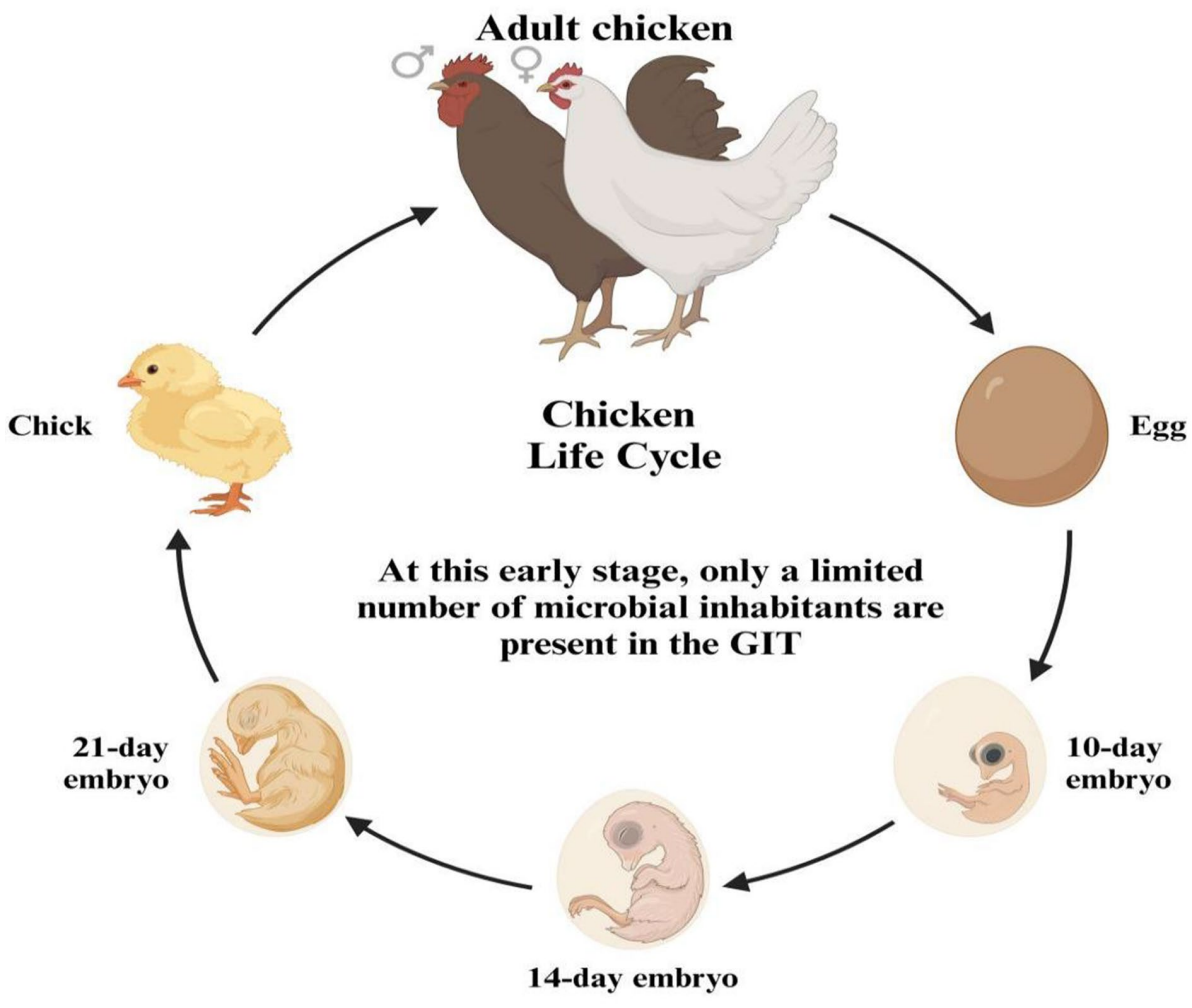
Early microbial colonization of the gut during the chicken life cycle.

**Figure 4:**
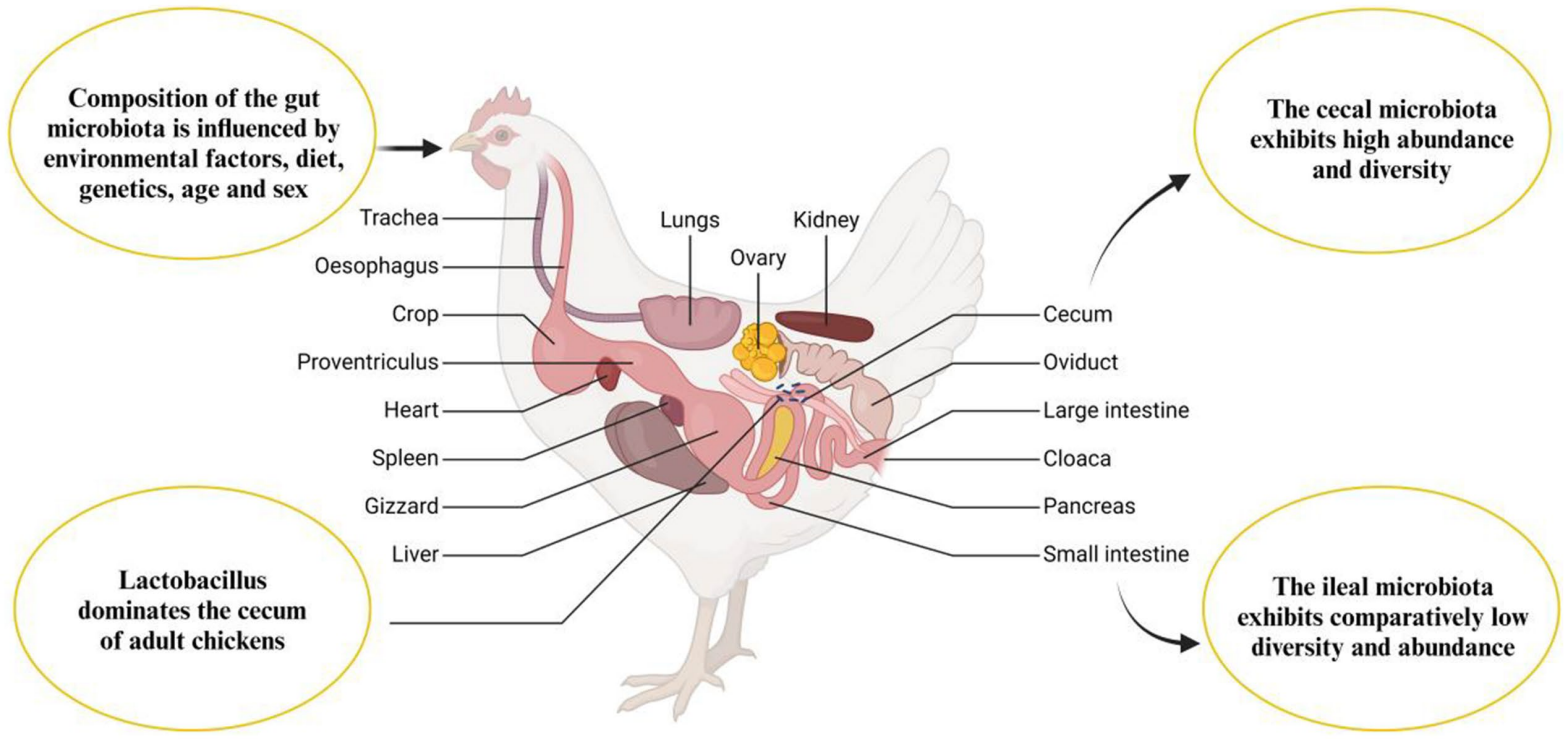
Anatomy of the chicken gastrointestinal tract and factors influencing gut microbiota composition.

The GIT of a newly hatched chick is nearly devoid of microorganisms, indicating a state close to sterility. In this early stage, only a limited number of microbial inhabitants are present in the GIT (Figure 3). These initial microbes are not randomly acquired from the environment but are instead vertically transmitted from the mother hen to her chicks, either through the oviduct or via the eggshell pores. This early microbial colonization plays a crucial role in the chick’s development and health by establishing a foundation for a more complex and diverse gut microbiome as the chick matures (30, 31). Additionally, microbiota can be transferred to the gastrointestinal tract during hatchery handling and transportation (32), and microbes have been detected in the chick’s GIT while it is still inside the shell (33). The early stage of the post-hatch microbial contamination affects the immune system and intestinal microbiota (34). The natural intestinal microflora develops after hatching and rapidly increases (35), from the 1st to the 19th day of life (36). The microbial colonization continuously grows until the GIT population reaches its balance (37). The fungi are more inhabited in the upper GIT site than the lower parts, while the bacterial inhabitance follows an opposite pattern (38). The microbiota of young chickens exhibits high variability (39–41) and defining the core chicken gut microbiota necessitates a focus on the microbiota of adult chickens, specifically those aged at least 20 weeks. This is due to the high variability and incomplete establishment of microbial communities in younger chickens. By studying adult chickens, researchers can discern the stable and consistent microbial populations that typify a well-developed gut microbiota. These matured adult chickens have undergone significant maturation, leading to a more stable and representative state of their gut microbiota, thereby offering a clearer understanding of the essential core microbial species for maintaining gut health and function (42–45). The microbial diversity within the gastrointestinal tract of chickens exhibits regional variations (Figure 4). *Lactobacilli* dominate the proximal parts of the digestive tract in adult chickens, although other species are also present (46, 47). Composition and complexity of the microbiota increase significantly in the distal segments of the intestinal tract, such as the cecum and colon. However, variations in colonic microbiota can be attributed to the chicken’s intestinal physiology and may exhibit similarities to either ileal or cecal microbiota.

### 3.2 Microbiota composition in the cecum

In the composition of the gut microbiota in chickens, bacteria represent the predominant group, constituting over 90% of the total microbial population. The most common bacterial groups include *Lactobacillus*, *Bacteroides*, *Bifidobacterium*, *Clostridium*, and *Bacillus cereus*, as well as *Escherichia coli*, yeast, and other fungi (48). The complexity and absolute numbers of gut microbiota significantly increase with age and environmental exposure. Most of these microbial species are classified into two major phyla, including the Gram-positive Firmicutes and the Gram-negative Bacteroidetes. The diversity and abundance of these groups highlight their essential role in digestion and maintaining gut health. Several studies emphasize the importance of a balanced microbial community within the cecum for optimal gut ecosystem function (49, 50).

In addition to Firmicutes and Bacteroidetes, two less dominant phyla, Actinobacteria (Gram-positive) and Proteobacteria (Gram-negative), are also present. In healthy adult hens, Firmicutes and Bacteroidetes typically represent about 45% each of the total microbiota, while Actinobacteria and Proteobacteria usually range from 2 to 3%. It is important to note that studies using 16S rRNA sequencing may slightly underestimate the abundance of Actinobacteria due to the low copy number of the 16S rRNA genes in species like *Olsenella*, *Collinsella*, and *Bifidobacterium* (51). While there is broad agreement on the overall composition of the cecal microbiota, significant individual variation persists. For instance, the proportion of Bacteroidetes can range from 10 to 90% without any signs of abnormality, and cases have been documented where chicks exhibit over 10% Actinobacteria and Proteobacteria. Despite variations in abundance, representatives from all four phyla are consistently found in the ceca of nearly all adult chickens.

### 3.3 Composition of small intestine microbiota

The small intestinal microbiome of chickens is a sophisticated and dynamic system, characterized by intricate interactions among numerous microorganisms. The bacterial composition in the small intestine is primarily dominated by four major phyla: Firmicutes, Bacteroidetes, Proteobacteria, and Actinobacteria. Among these, Firmicutes, represented by genera such as *Lactobacillus* and *Clostridium*, typically dominate. These bacteria can act either as symbionts or pathogens, depending on the strain type (52–54). The composition of the microbiota in the different sections of the small intestine, such as the duodenum, jejunum, and ileum, shows a high degree of similarity, as indicated by previous studies (45, 55). However, further comprehensive research is necessary to precisely identify the bacterial species uniquely adapted to each specific compartment of the small intestine. Occasionally, the microbiota of the ileum can intermingle with microorganisms originating from the caecum (55).

Firmicutes remain the most prevalent phylum in the small intestine, with genera such as *Lactobacillus*, *Enterococcus*, *Turicibacter*, *Clostridium sensu stricto*, as well as isolates from the *Clostridium* cluster within the family *Peptostreptococcaceae*, and the genus *Romboutsia*. In addition to Firmicutes, bacteria from the phylum Proteobacteria, including *Escherichia coli* and *Helicobacter*, can also be found in the small intestine. Notably, the presence of *Helicobacter* has been associated with reduced performance in chickens (56).

Moreover, despite the diversity of microbial species, the small intestine’s microbiota demonstrates limited diversity, with one to five genera accounting for around 50% of the entire ileal microbiota. This suggests a simpler microbial structure compared to other regions of the gastrointestinal tract (56). The ileum, a crucial component of the chicken’s digestive system, plays an essential role in nutrient absorption and immune regulation. It harbors a diverse microbial community that contributes significantly to maintaining intestinal health, enhancing nutrient absorption, and protecting against pathogens. These microorganisms break down complex carbohydrates, proteins, and lipids to produce short-chain fatty acids such as acetic acid, propionic acid, and butyric acid. These fatty acids not only provide energy for intestinal epithelial cells but also improves feed efficiency by promoting sodium and water absorption (40, 57, 58).

### 3.4 Fecal microbial composition

Many scientific studies utilize fecal samples to characterize the microbiota of chickens (46, 55, 59, 60). When experiments require repeated sampling from the same bird, researchers must often rely on fecal material. However, several considerations must be addressed during sample collection. First, it is difficult to prompt each chicken to defecate on command. Consequently, researchers frequently collect fecal material directly from the floor, making it impossible to control how long the droppings were exposed to air, whether for 10 min or 5 h. This is important because many gut colonizers are strict anaerobes, and exposure to air may reduce their viability or alter their community structure, potentially influencing the study’s final results.

Apart from environmental exposure, chicken digestion physiology significantly impacts the composition of colonic and fecal microbiota, chickens have a remarkably short digestive transit time, with digesta passing from ingestion to excretion in as little as 2 h (61, 62). Unlike mammals such as pigs or humans, adult chickens have a relatively short colon, measuring only about 10 centimeters, which limits the retention time of digesta. After stomach processing, most digesta quickly moves from the small intestine to the colon and is excreted approximately every 2 h (63). In contrast, only a small portion of the digesta moves from the ileum to the caecum, where it undergoes fermentation, for 8 to 12 h (64, 65). This rapid transit and short retention time shape the microbial composition found in the colonic and fecal matter, which must be considered when interpreting microbiota data. Additionally, the periodic voiding of caecal contents into the colon, which typically occurs twice daily, adds another layer of variability to sample composition (66, 67). If samples are collected immediately after caecal voiding, the colonic or fecal microbiota may closely resemble the caecal microbiota. Conversely, if samples are collected before the next caecal voiding, the microbiota may more closely resemble the ileal community. When digesta from the small intestine passes through the colon after caecal excretion, the microbiota could represent a mixture of both caecal and ileal communities. Thus, variations in the timing of sample collection related to periodic caecal voiding and intestinal transit contribute to substantial variability in colonic or fecal microbial composition (55, 59, 68, 69), and researchers must account for this in their experimental designs.

### 3.5 Major bacterial taxa colonising chicken intestinal tract

#### 3.5.1 Classification of intestinal bacteria at the major phyla

The gut microbiome consists of bacteria classified across various taxonomic ranks, including phylum, class, order, family, genus, and species. These bacteria exhibit distinct distribution patterns and perform a range of functions in different segments of the intestinal tract. Among the major bacterial phyla, Bacteroides predominate in the chicken cecum (Table 1). These bacteria are well-known for their ability to degrade complex polysaccharides and plant cellulose, engaging in fermentation processes that produce short-chain fatty acids such as acetic acid, propionic acid, and butyric acid. These fatty acids play a crucial role in maintaining intestinal acid–base balance and inhibiting pathogen proliferation (69). It should be noted that Bacteroides are involved in vitamin synthesis and bile acid metabolism, both of which are essential for nutrient absorption and host metabolism (70). Additionally, Firmicutes represent a significant group of anaerobic bacteria in the chicken gut, including a substantial number of *Clostridium* species. While some *Clostridium* species can produce potentially harmful metabolites, they also contribute to cellulose degradation and short-chain fatty acid production (71). Especially, certain Firmicutes bacteria possess probiotic properties, helping to modulate the intestinal microecological balance and enhance the host’s immune response (72).

**Table 1:** The primary role of the core microorganism.

Gut microbiota		Effects on the gastrointestinal tract	References
Phylum	Firmicutes	Engaged in the synthesis of vitamins and the metabolism of bile acids	Flint et al. (70)
	Bacteroidetes	Promote cellulose degradation and short-chain fatty acid production	Louis and Flint (71)
	Actinobacteria	Improve the digestibility of feed	Lewin et al. (142)
Genus	*Campylobacter*	Invasion of the intestinal epithelial cells leads to damage in the intestinal mucosa	Yan et al. (55) and Han et al. (74)
	*Helicobacter*		
	*Clostridium*	Generate butyrate through the conversion of acetyl-CoA	Medvecky et al. (76)
	*Blautia*	In scavenging free hydrogen released by numerous anaerobic bacteria during fermentation	Sergeant et al. (77)
	*Lactobacilli*	Improved efficiency in the fermentation of carbohydrates	Crhanova et al. (75)
	*Lactobacillus agilis*	The organism possesses genes responsible for encoding the flagella’s structure	Eeckhaut et al. (143)
	*Ruminococcaceae*	Production of butyrate occurs	Esquivel et al. (144)
Family	Lachnospiraceae	Produce various short-chain fatty acids, such as acetate, propionate and succinate	Polansky et al. (73)
	Porphyromonadaceae	Each family member possesses genes that encode enzymes like methylmalonyl epimerase, mutase, and decarboxylase. These enzymes facilitate the biochemical conversion process, enabling the production of propionate from succinate	Adamberg et al. (80) and Isar et al. (81)

#### 3.5.2 Classification of intestinal bacteria at the major generic

Actinobacteria are a group of Gram-positive bacteria with high GC content (around 65%), playing a key role in the breakdown of organic matter and are known for their production of secondary metabolites such as antibiotics, enzymes, and other bioactive compounds. In the chicken cecum, the predominant genera of colonizing bacteria include Coriobacteriaceae, represented by *Olsenella* and *Collinsella* from the Bifidobacteraceae family, which also includes *Bifidobacterium*. Proteobacteria, which are non-spore-forming, Gram-negative bacteria, are also present. Common colonizing bacteria in the chicken cecum include facultative organisms like *E. coli*, as well as *Desulfovibrio*, *Sutterella*, *Parasutterella*, *Anaerobiospirillum*, and *Succinatomonas*. Additionally, Actinomycetes contribute to poultry health by producing nutrients and metabolizing short-chain fatty acids, enhancing digestive efficiency and improving feed conversion rates (73).

*Helicobacter* and *Campylobacter* are also prevalent components of the chicken microbiota. In highly infected chickens (55, 74), *E. coli* and *Salmonella* constitute approximately 0.1% of the total microbiota, while *Campylobacter* and *Helicobacter* can account for over 10%. This highlights differing colonization patterns between *E. coli*, *Salmonella*, and bacteria like *Helicobacter* and *Campylobacter*. Other bacterial families in the cecum include *Lactobacillaceae*, *Veillonellaceae*, and *Erysipelotrichaceae*. *Trichiidae*, which consist of strictly anaerobic spore-forming bacteria with a genomic GC content of about 45%, produce butyrate from acetyl-CoA. Notable species include *Bacillus coli*, *Clostridium lactofermens*, and *Clostridium saccharolytica*, which are significant butyrate producers (75, 76).

*Cyanobacteria* contribute to microbial metabolism by encoding enzymes like 5-methyltetrahydrofolate: cobalamin methyltransferase and acetyl-CoA synthetase (73). These enzymes enable Cyanobacteria to use CO_2_ and H_2_ to generate acetic acid through reductive acetogenesis (77). Similarly, *Blautia* plays a key role in scavenging free hydrogen released by anaerobic bacteria during fermentation.

*Lactobacilli* efficiently ferment carbohydrates (78), producing lactic acid and lowering the environmental pH. This acidic condition inhibits the growth of other bacterial species, giving *Lactobacilli* a competitive advantage in diverse environments, including the gastrointestinal tract and fermented foods. Certain strains, like *Lactobacillus ruminis* and *Lactobacillus agilis*, possess flagella, enhancing their motility and ability to colonize and acquire nutrients in complex environments, such as the gut (78, 79).

*Ruminococcaceae* are primary butyrate producers. Through carbohydrate fermentation, most *Ruminococcaceae* convert two acetyl-CoA molecules to crotonyl-CoA, resulting in butyrate production (73). Flavonoids and pseudoflavonoids can also produce butyrate through lysine fermentation or succinic acid reduction. *Anaerotruncus* represents a potentially mobile intestinal colonizer. Due to their high sensitivity to oxygen, *Ruminococcaceae* and *Lachnospiraceae* are often among the first bacterial families to diminish during inflammatory diseases, as reactive oxygen species from macrophages and granulocytes damage the gut microbiome. Therefore, the reduction of *Ruminococcaceae* and *Lachnospiraceae* is typically a consequence, rather than a causative factor (78, 79).

#### 3.5.3 Taxonomic classification of predominant bacterial families in the intestines

The family characteristics frequently observed in the chicken cecum include members of the Bacteroidaceae, as well as Gram-positive families, like Lachnospiraceae and Ruminococcaceae. The genus *Bacteroides* comprises numerous species that show host-specific adaptations. For instance, human-adapted species such as *B. dorei*, *B. uniformis*, and *B. clarus* contrast with chicken-associated species like *B. salanitronis*, *B. caecigallinarum*, and *B. coprocola*. Interestingly, although not yet fully understood, certain chicken-associated *Bacteroides* strains have acquired KUP genes encoding potassium ion transporters. In adult chickens, Bacteroidaceae genomes contain a high number of genes responsible for the degradation of complex polysaccharides. These bacteria produce short-chain fatty acids (SCFAs) such as acetate, propionate, and succinate. Notably, *in vitro*, *Bacteroides* species show nearly the same acidification capability as *Lactobacilli*, which are well-known for their acid production (Table 1). As a result, the extensive fermentation by *Bacteroides* species significantly contributes to the acidic environment in the caecum and plays a critical role in digestion (73, 76).

The phylum Bacteroidetes includes several distinct families, such as Rikenellaceae, Bacteroidaceae, Prevotellaceae, and Porphyromonadaceae. Bacteroidetes genomes are relatively large, ranging from approximately 3 to over 6 megabase pairs (Mbp). Members of this phylum possess genes encoding enzymes like methylmalonyl-CoA epimerase, mutase, and decarboxylase, which are crucial for the biochemical conversion of succinate to propionate (80–82).

The Rikenellaceae and *Alistipes* stand out with a GC content of 58–60%, distinguishing them from other members of Bacteroideae. Remarkably, *Alistipes* is one of the earliest colonizers of the chick cecum within the Bacteroidetes group (83, 84). The Porphyromonadaceae family consists of genera such as *Barnesiella*, *Odoribacter*, *Butyricimonas*, and *Parabacteroides*. Both *Bacillus putitidis* and *Bacillus butyricomonas* can produce butyrate through lysine fermentation and succinic acid reduction pathways. Additionally, butyrate can be synthesized from acetyl-CoA (76).

Prevotellaceae is primarily responsible for the degradation of cellulose and other complex carbohydrates. Members of this family are renowned for their diverse and intricate metabolic capabilities (83, 85). The characteristics of chicken isolates from the Prevotellaceae family remain largely unexplored. The isolates obtained in pure culture show only distant relations to characterized Prevotella species, with 16S rRNA sequences displaying about 90% similarity to the closest GenBank entries. This suggests that the Prevotella family in chickens may represent new genera distinct from those in humans, mice, or pigs. Omics studies have shown that chicken Prevotellaceae specialize in digesting complex polysaccharides (75), paralleling the presence of Prevotellaceae in the rural African gut microbiota (84, 86).

## 4 Gut microbiota and host interactions

### 4.1 Pathogen colonization interacts with the gastrointestinal tract

Bacteria pathogenic to poultry include species such as *Staphylococcus*, *Escherichia coli*, *Clostridium*, *Campylobacter*, and *Salmonella* spp. (49). One of the most notable pathogens is *Salmonella*, a bacterial threat in poultry that presents significant risks for human health due to its prevalence and potential for contamination (87, 88). *Salmonella* can easily colonize the intestines of poultry. However, research is underway to explore the use of targeted bacteriophages to eliminate *Salmonella* from the microbiome of broiler chickens (89). Among the various strains, *Salmonella typhimurium* is particularly concerning for humans, as it can colonize the poultry digestive system without causing harm to the birds themselves (90). Fortunately, current research focuses on microbial interventions, such as probiotics and metabolites, to prevent *Salmonella* colonization and promote the restoration of a healthy gut microbiota after infection (90–92).

Another pathogen that affects the poultry intestines is *Clostridium perfringens* (93), particularly types A and C, which can lead to necrotic enteritis (NE), characterized by decaying and inflamed intestinal tissue. This condition generally arises under specific dietary conditions, often in conjunction with the presence of the parasite coccidia (93, 94). In addition to intestinal pathogens, respiratory infections (RI) in poultry are also a concern. A 2021 study on the bacterial composition of the turkey respiratory tract revealed that the presence of *Ornithobacterium* and *Mycoplasma* increases the risk of RI in turkeys (95). Moreover, domesticated turkeys were found to have less microbial diversity and a higher prevalence of pathogenic and antibiotic-resistant bacterial strains compared to their wild counterparts (96).

### 4.2 Nutritional role of the chicken gut microbiome and host

#### 4.2.1 Fermentation and the production of short-chain fatty acids

**Figure 5:**
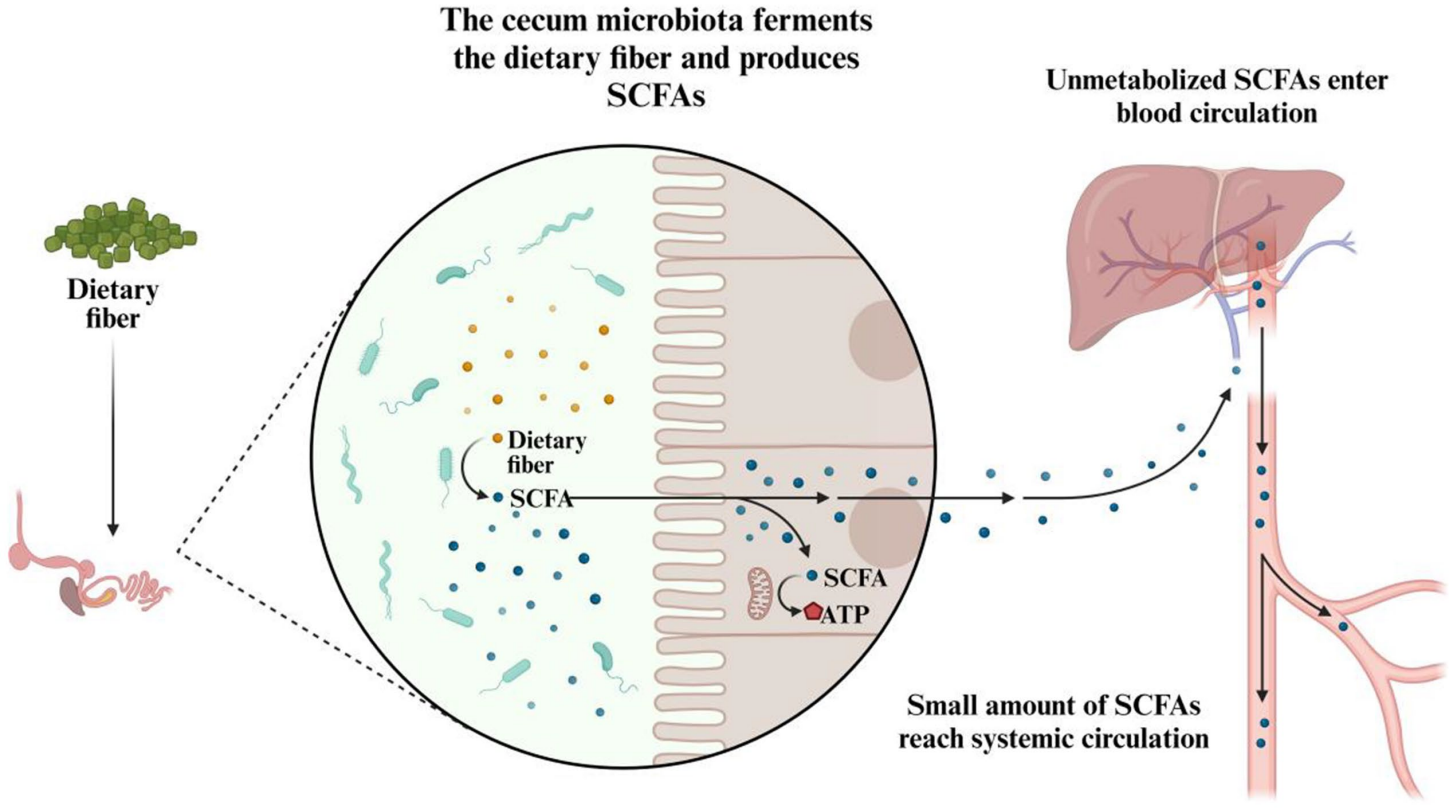
The role of cecal microbiota in the fermentation of dietary fiber and production of SCFAs.

A key function of the chicken gut microbiome is the fermentation of dietary fibers and other complex carbohydrates (Figure 5). Since chickens lack the necessary enzymes to break down certain fibers found in plant-based feeds, their gut microbiota compensates by fermenting these substrates in the cecum and colon. This fermentation process produces short-chain fatty acids (SCFAs), such as acetate, propionate, and butyrate (97), which are crucial energy sources for chickens. For example, acetate is absorbed into the bloodstream and serves as an energy substrate for various tissues, including muscles, and the liver (98). In contrast, propionate and butyrate are mainly utilized by the gut epithelium, with butyrate playing a particularly critical role in maintaining intestinal cell health, enhancing gut barrier function, and reducing inflammation (99). Besides providing energy, these SCFAs also contribute to the structural integrity and immune function of the gut, which are vital for overall health and efficient nutrient absorption.

It is important to emphasize that dietary carbohydrates are broken down and absorbed primarily within the proximal gastrointestinal tract, while undigested carbohydrates, along with residual digestible ones, proceed to microbial fermentation in the distal gastrointestinal tract (100). The gut microbiota can hydrolyze non-digestible disaccharides, oligosaccharides, and polysaccharides into their monosaccharide components through fermentation, producing SCFAs, which are then used by the host for energy generation and as a carbon source (101, 102). This fermentation activity predominantly occurs in the ceca, where the microbial density is highest (103), and increases as the chicken matures. While absent in the ceca of one-day-old chicks, optimal SCFA concentrations are typically achieved by day 15 post-hatch and remain stable afterward (104). Through passive diffusion across the cecal epithelium, SCFAs enter various metabolic pathways. Research has also highlighted the regulatory roles of SCFAs, which include modulating colonic blood flow, stimulating enterocyte development and proliferation, enhancing mucin production, and regulating the intestinal immune response (105, 106).

#### 4.2.2 Vitamin interaction

The gut microbiota of chickens plays a crucial role in the complex biosynthesis of various B vitamins, such as biotin, folate, and riboflavin. Research has demonstrated that specific bacterial species within the chicken gut can produce these vitamins through fermentation processes (107). For instance, *Lactobacilli* and *Bifidobacteria* are capable of producing folate and biotin, which are essential for cellular functions and metabolism (107, 108). However, certain gut microbes can degrade vitamins, potentially leading to deficiencies. For example, some strains of *Clostridium* spp. have been shown to degrade vitamin B12, reducing its availability to the host (109). This degradation may result in vitamin deficiencies and subsequent health issues in chickens. Additionally, the gut microbiota influences the absorption of fat-soluble vitamins, such as vitamins A, D, E, and K. Specific microbial populations can either enhance or inhibit the absorption of these vitamins. For example, a study found that a diverse gut microbiota improves the absorption of vitamins A and E by altering bile acid metabolism and strengthening intestinal barrier function (110). Deficiencies or imbalances in vitamins due to microbial activity can lead to poor growth rates, immune dysfunction, and increased susceptibility to diseases (109).

#### 4.2.3 Protein metabolism

Metabolism of proteins in chickens is a crucial component of their nutritional needs and growth requirements. The gut microbiota play a significant role in this process by influencing protein digestion, amino acid absorption, and the generation of bioactive peptides. Although the digestion of dietary proteins begins in the stomach and small intestine, a substantial portion of protein digestion and fermentation occurs in the cecum and colon, where the gut microbiota are abundant. These microbial communities assist in breaking down proteins that the host’s enzymes cannot fully digest.

**Figure 6:**
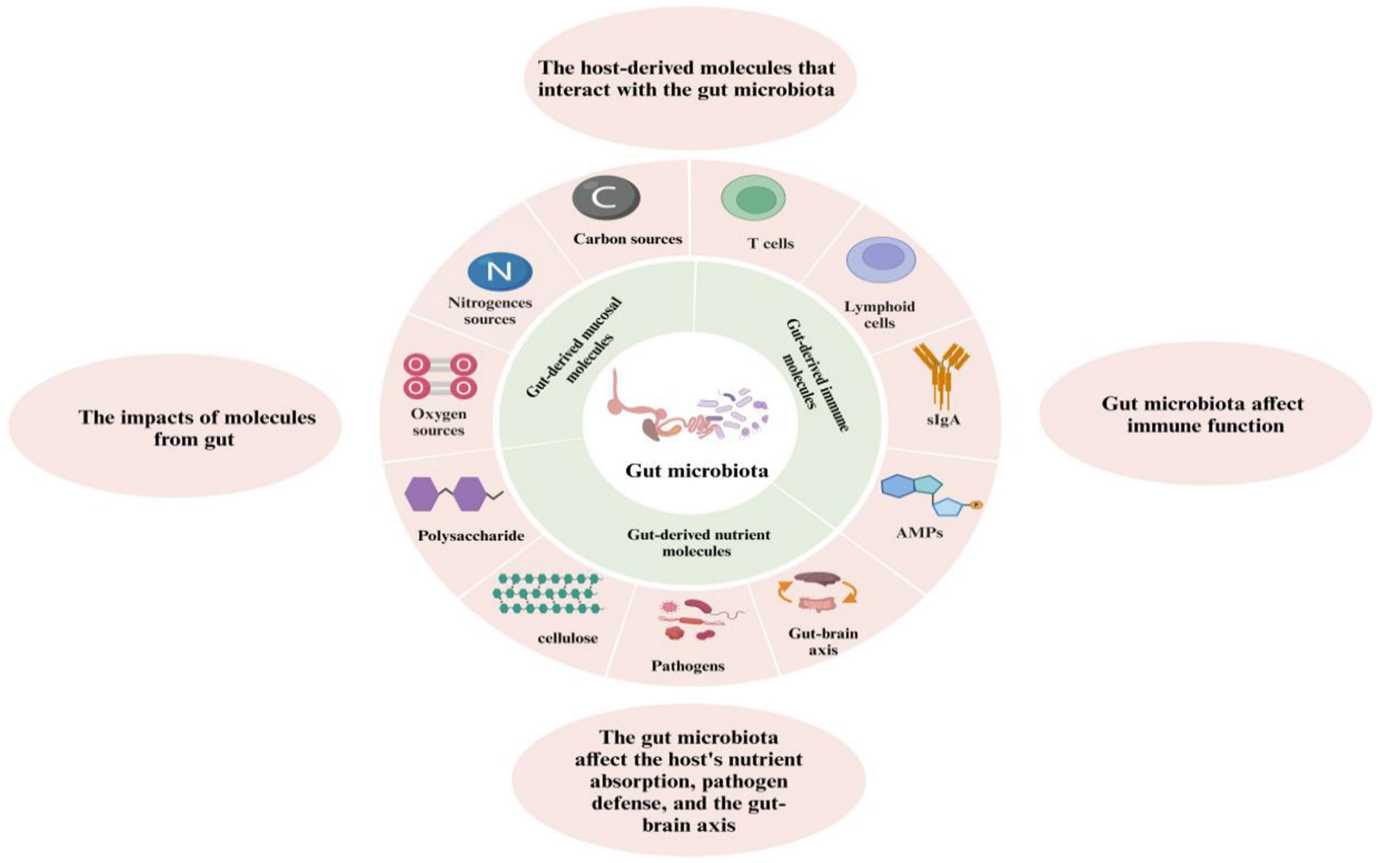
The role of gut microbiota in host interactions: metabolism, immunity, and the gut-brain axis.

Proteolytic bacteria in the gut ferment dietary proteins into various peptides and amino acids (107). For instance, bacteria such as *Clostridium* and *Lactobacillus* produce enzymes that degrade proteins into shorter peptides and free amino acids. This microbial proteolysis provides an essential secondary mechanism to break down proteins that escape digestion in the upper gastrointestinal tract (111). The amino acids released through microbial protein degradation are absorbed by the host and utilized in various metabolic processes (Figure 6). Gut microbes affect the availability of these amino acids by influencing their absorption and conversion. Some microbial enzymes can release amino acids bound in dietary proteins or peptide bonds that resist degradation by host enzymes. For example, *Bacteroides* and *Bifidobacteria* contribute to protein hydrolysis and amino acid release, which the host’s intestinal cells subsequently absorb (112).

In addition to aiding digestion, gut microbes produce bioactive peptides that exert physiological effects on the host. These peptides can influence immune responses, gut health, and growth. Some of these microbial-derived peptides possess antimicrobial properties or modulate immune functions. For instance, certain peptides produced by gut bacteria have been shown to enhance the host’s immune system and protect against pathogenic infections (109). Furthermore, efficient microbial protein digestion and amino acid absorption promote improved growth rates and better feed conversion ratios. Research indicates that maintaining a balanced gut microbiota optimizes protein utilization, enhancing feed efficiency. For example, beneficial microbes such as *Lactobacillus* improve protein digestibility while reducing the production of nitrogenous waste, leading to better growth outcomes and improved feed conversion (112).

#### 4.2.4 Mineral absorption

Composition of gut microbiota impacts the absorption of essential minerals, including calcium, phosphorus and magnesium, by modulating the intestinal microenvironment. Beneficial bacteria, in particular, can produce short-chain fatty acids (SCFAs) through the fermentation of dietary fibers, which enhances the solubility and uptake of these minerals. Additionally, certain gut bacteria can biosynthesize vitamins and other compounds that further facilitate mineral assimilation (113). The presence of specific microbial species is linked to increased mineral bioavailability. Research shows that probiotic strains positively impact the availability of calcium and phosphorus in the gastrointestinal tract, leading to improved mineral absorption and utilization (114). In contrast, dysbiosis, or an imbalance in gut microbiota, can impair mineral absorption and cause deficiencies. Diets high in fiber and prebiotics promote a diverse and beneficial microbiota, which in turn optimizes mineral absorption. For example, dietary fibers encourage the growth of beneficial bacteria that produce SCFAs, thereby aiding in mineral absorption (115).

### 4.3 Modulation of the chicken immune system by gut microbiota

The development and function of gut-associated lymphoid tissue (GALT) in chickens are strongly influenced by the gut microbiome. A well-balanced microbiome strengthens the body’s defense mechanisms against threats and promotes a robust immune response (116). Additionally, this balance helps prevent excessive immune reactions to harmless antigens, such as food or commensal bacteria, thus protecting against unnecessary immune activation. Conversely, when microbial balance is disrupted, a condition known as dysbiosis (117), it can trigger inflammatory responses, increasing the risk of autoimmune disorders and making chickens more vulnerable to infections.

The colonization of beneficial bacteria promotes immune tolerance, effectively training the immune system to differentiate between pathogenic and non-pathogenic stimuli. This symbiotic relationship enhances the body’s capacity to mount efficient responses to pathogens while mitigating unnecessary immune reactions (118). Moreover, the gut microbiota influences the production of immune cells, such as macrophages, dendritic cells, and T cells, by releasing microbial metabolites (Figure 6). These metabolites act as signaling molecules that modulate immune responses both locally in the gut and systemically throughout the body. Research suggests that gut microbiota can enhance the production of secretory immunoglobulin A (sIgA) in the gut mucosa (119). sIgA is a key component of the immune system that serves as the first line of defense by neutralizing pathogens and toxins before they cross the epithelial barrier. Continuous production of sIgA is essential for protecting chickens from gastrointestinal infections, especially during periods of stress or exposure to pathogens. Dysbiosis not only compromises the gut’s physical barrier but also impairs the immune system’s ability to defend against infections. This disruption increases the risk of diseases such as necrotic enteritis, coccidiosis, and other gastrointestinal disorders that are common in poultry (119).

### 4.4 Gut microbiota mediated protection against viral diseases

Recent research highlights the essential role of gut microbiota in shaping immune responses to viral infections in chickens. Commensal bacteria contribute to pathogen defense through direct competition, antibody production, and the activation of cytokines, which modulate both innate and adaptive immune responses (120). However, several significant viral diseases can disrupt the diversity of the intestinal microbiota, leading to dysbiosis, a state associated with various pathological conditions that facilitate acute viral infections in chickens.

The gastrointestinal tract’s highly dynamic environment presents opportunities for pathogens to upset the balance between the host and microflora, resulting in dysbiosis and subsequent mucosal infections (121). Bacterial dysbiosis has been linked to inflammation and alterations in immune functions (122), particularly affecting type I interferons (IFNs) and inflammatory responses (123, 124). Diseases that destabilize intestinal microflora (125–127) further increase chickens’ vulnerability to bacterial infections during dysbiosis (128). Moreover, research has established links between gut microbiota and distant organs, forming key pathways such as the gut-lung, gut-brain, gut-skin, and gut-liver axes (129). In particular, species such as *Bifidobacterium*, *Firmicutes*, *Faecalibacterium*, *Blautia*, and *Clostridium* are essential for preventing and managing viral diseases. They outcompete pathogens and colonize the gastrointestinal mucosal surface, thereby maintaining microbial balance.

These beneficial microbiota also support digestion and produce short-chain fatty acids (SCFAs), which serve as an energy source and modulate antiviral immune responses. SCFAs stimulate the production of interferons (IFN-α and IFN-β) and enhance the function of T regulatory cells. This process promotes the secretion of anti-inflammatory cytokines such as IL-22 and strengthens humoral immune responses through the production of IgA and IgG antibodies. Together, these mechanisms contribute to controlling the severity of viral infections in chickens (130–132).

### 4.5 Interaction between gut microbiota and the gut-brain axis

The gut-brain axis (GBA) serves as a bidirectional communication network that connects the gut microbiota with the central nervous system (CNS). The gut microbiota can influence the production of neurotransmitters, which act as chemical messengers transmitting signals between the gut and the brain (Figure 6). Specific gut bacteria, for example, have the ability to produce neurotransmitters like serotonin and gamma-aminobutyric acid (GABA), which play crucial roles in mood regulation and behavior. Studies conducted on chickens have demonstrated that changes in the composition of gut microbiota can affect levels of these neurotransmitters, thereby influencing their overall well-being and stress responses (133). The modulation of behavioral responses in chickens is closely linked to the functioning of the gut-brain axis. Changes in gut microbiota can impact stress levels, anxiety, and behavior, suggesting that the gut microbiota, through its connection with the gut-brain axis, may modulate behavioral traits, potentially affecting productivity and welfare in poultry (134). Additionally, alterations in immune function due to changes in short-chain fatty acids (SCFAs) produced by gut bacteria can affect brain health by interacting with inflammatory cytokines and influencing neuroinflammation. In chickens, dysregulated microbial communities may lead to increased intestinal permeability and systemic inflammation, potentially exerting negative effects on brain function and behavior (135). Notably, stress responses are influenced by the gut microbiota, and stress itself can alter the microbial composition, creating an imbalanced state that worsens the stress response and affects overall health. Variations in microbial communities have been associated with changes in stress hormone levels and intestinal integrity, all of which contribute to how chickens respond to stress and influence their general health condition (136). Unfortunately, the relationship between the gut microbiota and serotonergic activity, as well as related psychological health, has not been thoroughly investigated.

The gut microbiota can also affect levels of neurotransmitters such as dopamine and serotonin. Certain gut bacteria participate in the production of these neurotransmitters or their precursors. For example, the microbiota can influence the synthesis of tryptophan and tyrosine, the amino acids that are precursors for serotonin and dopamine, respectively (137). Alterations in gut microbiota composition can lead to changes in neurotransmitter levels, which may, in turn, affect behavior and stress responses in chickens. Imbalances in the gut microbiota can result in decreased serotonin levels, potentially impacting mood and increasing stress (138). Changes in dopamine levels may also affect motivational and reward-related behaviors. Research indicates that dietary changes influencing the gut microbiota can modulate these neurotransmitter levels, thereby altering behavioral responses in chickens (139). Moreover, the gut microbiota can affect the function of the blood-brain barrier and modulate levels of neurotransmitters like dopamine and serotonin, impacting cognitive functions and emotional states (140). It should be noted that imbalances in gut microbiota affecting neurotransmitter levels can contribute to stress, anxiety, and other health issues (141). Understanding these interactions can help in formulating dietary and management strategies to maintain a healthy gut microbiota and promote an optimal neurotransmitter balance, ultimately enhancing the well-being of chickens.

## 5 Conclusion

Although advances in biotechnology have improved our understanding of the poultry microbiome and its role in poultry health and disease, further exploration of the GIT ecosystem, including the factors that influence it, and the interactions between the host and microbiome in chickens, is necessary to fully grasp the dynamics at play. This review aims to enhance our understanding of intestinal ecosystems while simultaneously establishing a robust foundation for the development of more effective strategies in the management and treatment of poultry diseases. A central focus of future research should be on investigating how changes in the composition of the poultry microbiome correspond to environmental factors, growth conditions and specific feed formulations. Additionally, it is essential to identify advantageous microbial strains that play a pivotal role in sustaining poultry health through the application of diverse methodologies. The potential of nanotechnology to enhance the nutritional value of dietary factors presents an exciting opportunity for improving poultry health. Lastly, integrating metagenomics and metabolomics approaches will advance the study of GIT diseases in poultry, offering a more holistic understanding of the relationship between the gut microbiome and overall health.
